# The role of multidisciplinary team discussions in the management of thoraco-omphalopagus conjoined twins: A case report from a low-resource setting

**DOI:** 10.1016/j.ijscr.2025.111920

**Published:** 2025-09-08

**Authors:** Brian Kasagga, Roy Clark Basiimwa, Yasin Ssewanyana, Stella Nimanya, Rita Nassanga, John Sekabira

**Affiliations:** a Department of Surgery, Mulago National Referral Hospital, Uganda; b Department of Radiology, Mulago National Referral Hospital, Uganda

**Keywords:** Conjoined twins, Thoraco-omphalopagus, Imaging, Case report, Multidisciplinary teams

## Abstract

**Introduction and importance:**

Conjoined twins represent a rare medical phenomenon that poses significant clinical and ethical challenges, particularly in resource-limited settings.

**Case presentation:**

We present a case of conjoined twins delivered at 39 weeks gestation to a 27-year-old refugee mother. The delivery was by emergency cesarean section due to footling breech presentation. Initial assessments revealed shared cardiac anatomy, with Twin A exhibiting a more favorable anatomical structure compared to Twin B, who presented with severe cardiac malformations. Follow-up imaging, including CT angiography, uncovered additional complexities, such as dextrocardia and situs inversus in Twin A, raising further challenges regarding surgical separation.

**Clinical discussion:**

Management required a multidisciplinary discussion involving social worker, nurses, pediatric surgeons, neonatologists, pharmacist, nutritionist, pediatric cardiac surgeons, radiologists, and anesthesiologists to address both clinical challenges and the ethical considerations surrounding separation in the context of one twin with unfavorable anatomy. The team emphasized tailored care and adjustment of medication dosages due to the shared circulatory system of the twins. Despite the team's efforts, both twins succumbed to heart failure.

**Conclusion:**

This case highlights the clinical and ethical complexities of managing conjoined twins with shared vital organs in a low-resource setting. It underscores the importance of advanced imaging, multidisciplinary planning, and social support;- especially for vulnerable populations like refugees. Strengthening antenatal care, including routine obstetric ultrasound, is essential for early diagnosis, timely referral, and improved outcomes.

## Highlights

• Management of conjoined twins poses unique challenges in a low-resource settings.
• Ethical dilemmas arise when one twin has more favorable anatomy than the other.
• Multidisciplinary team work is essential in thoraco-omphalopagus case management.

## 1 Introduction

Conjoined twins are a rare congenital condition resulting from incomplete splitting of a single fertilized egg during early embryogenesis, typically around the third week of gestation [1]. The incidence ranges between 1 in 50,000 and 1 in 200,000 live births [2]. Epidemiological studies indicate that conjoined twins are more common in Asian and African communities and are three times more likely to occur in females [2]. The earliest well-documented case of conjoined twins is that of Chang and Eng Bunker, born in 1811 in Siam (modern-day Thailand), whose condition later coined the term “Siamese twins” [3,4].

While rare globally, conjoined twinning presents unique ethical, diagnostic, and surgical challenges, particularly in low-resource settings where access to advanced imaging, neonatal intensive care, and multidisciplinary expertise is limited [5]. In Uganda, the earliest published reports by Zake et al. in a 10-year period (1971–1980). Sixteen sets of conjoined twins were delivered at a regional referral hospital, with an incidence of 1 in 4242 deliveries, 1 in 69 multiple births, and 1 in 139 congenitally malformed babies [6]. More recently, Ugandan surgical teams successfully operated two pairs of conjoined twins in 2021 and 2022 at a tertiary hospital and a regional referral hospital [7].

Due to limited surveillance systems and reporting, the true prevalence of conjoint twins remains unclear particularly in sub–Saharan Africa [2]. Nearly half of all conjoined twins are stillborn, and up to a third of those delivered alive die within 24 h [8,9]. For those who survive the first 24 h, separation if feasible requires substantial preoperative planning. This includes time for weight gain, anatomical mapping of shared organs, multidisciplinary discussions, mobilizing financial resources and family case conferences [[10], [11], [12]]. Preoperative radiological assessment is essential in the management plan providing anatomical mapping of shared organs and structures [13,14]. This allows for precise surgical planning and increasing the likelihood of successful separation [15].

We present a case of thoraco-omphalopagus conjoined twins, referred from a lower facility after birth to a tertiary hospital for further management. We highlight how multidisciplinary team discussions played a crucial role in managing this case.

## 2 Methods

This case report has been reported in line with the SCARE criteria (Surgical CAse REport) 2025 guidelines, ensuring comprehensive and transparent reporting of the clinical details [16].

## 3 Case presentation

The mother delivered the twins by cesarean section at a Health Centre III facility in the mid-western region of Uganda, located within a refugee settlement. She was unaware of the twin gestation or the conjoined nature of the pregnancy until delivery. The facility is a Health Centre III serving a refugee population of approximately 180,000–240,000 individuals from conflict-affected regions of South Sudan and the Democratic Republic of Congo.

Living conditions in the settlement are often overcrowded, with limited access to clean water, basic infrastructure, and antenatal imaging services. Following delivery, the mother and newborns were referred to our institution, a public, tertiary academic referral hospital in Uganda that serves as a national centre for complex pediatric surgical care. The hospital has previously undertaken successful surgical separations of conjoined twins. It houses a dedicated pediatric surgery unit staffed by seven pediatric surgeons and supported by neonatologists, with a 30-bed capacity. Postoperatively, children are managed in a four-bed pediatric intensive care unit (PICU) equipped for mechanical ventilation and staffed by a dedicated critical care team. The hospital is adjoined to the Uganda Heart Institute, which performs complex cardiac surgeries and includes pediatric cardiac surgeons who actively participate in multidisciplinary case management.

The identical twins were delivered at term at 39 weeks by a prime gravida via emergency cesarean section due to breech presentation with footling. Preoperative assessments of random blood sugar levels and blood pressure were normal. There was no maternal fever, although the amniotic fluid was meconium-stained. The mother had no history of chronic illnesses such as hypertension or diabetes. She also had no history of familial/genetic diseases in her family or a history of conjoined twins in her family. She reported no alcohol intake, smoking, or use of illicit drugs. She attended antenatal care four times at a health center. Her HIV serology was negative, but blood grouping, rubella, Hepatitis B, and TPHA for syphilis tests were not performed. She did not undergo any ultrasound scans during her pregnancy due to equipment breakdowns at the health centre and the absence of reliable electricity. Notably, she suffered five episodes of malaria during her pregnancy, which were treated with oral antimalarial tablets (artemether/lumefantrine).

At birth, the Apgar scores for both twins were 3, 6, and 8 at the 1st, 5th, and 10th minutes, respectively. The combined birth weight was 5.7 kgs. They were appropriately resuscitated using bagging with oxygen. Intravenous antibiotics (ampicillin and gentamicin) were administered, along with Dextrose 10 %. They were then referred for further management of respiratory distress. The neonates were transported with their mother in an ambulance on oxygen, with SpO2 levels of 90 % for Twin A and 89 % for Twin B, on nasal prongs connected to an oxygen cylinder. The twins passed urine and meconium during transit within the first 24 h.

**Fig. 1:**
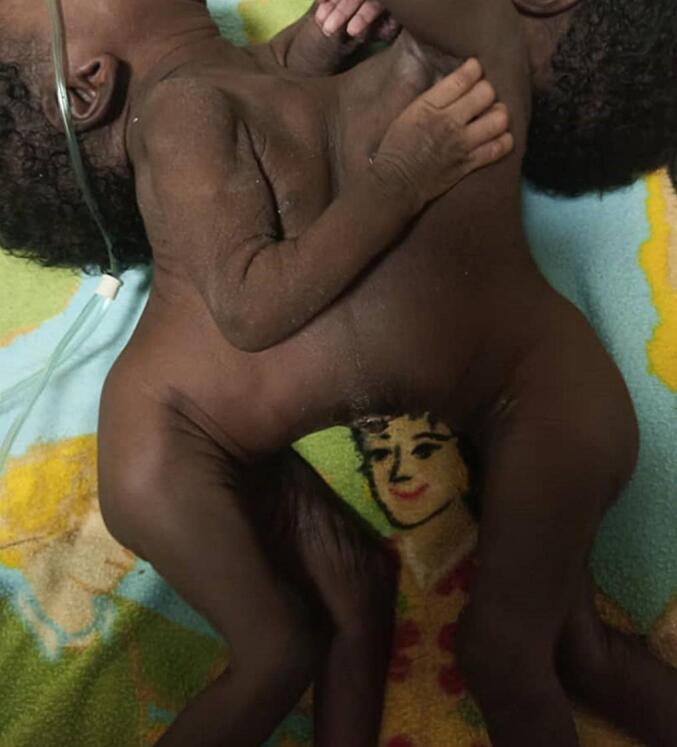
General examination showing twins joined at thorax and abdomen.

On examination, the twins were joined at the abdomen and thorax as seen in Fig. 1. Twin A (the larger twin) was afebrile with a temperature of 36.6 °C. She showed no signs of pallor, jaundice, or dehydration. Although tachypneic, her SpO2 was 99 % on 0.5 l of free-flow oxygen. Her chest was clear on auscultation. She had tachycardia with a heart rate of 171 bpm, but heart sounds were normal. Muscular tone and reflexes in her limbs were normal. Her abdomen was soft, and a digital rectal examination (DRE) confirmed a patent anus.

Twin B (the smaller twin) was also afebrile with a temperature of 36.7 °C. She showed no signs of jaundice, edema, dehydration, or pallor. Although tachypneic, her SpO2 was 98 % on free-flow oxygen. Her chest was clear on auscultation. She had tachycardia with a heart rate of 162 bpm, and heart sounds were normal. She had a smaller abdominal domain compared to her twin. Muscular tone and reflexes in her limbs were normal. A digital rectal exam (DRE) confirmed a patent anus, with meconium present on the examining finger.

### 3.1 Investigations

#### 3.1.1 Echocardiogram

Complex heart anatomy with a shared pericardial sac and interventricular septum. Twin A had well-formed heart chambers, small patent Foramen ovale (PFO), small patent ductus arteriosus (PDA), and small mid-muscular ventricular septal defect (VSD). Twin B had slightly hypoplastic chambers, with a large PDA and separate pulmonary arteries and aorta. The findings of the echocardiogram are detailed in the table below;

**Table:** 

Twin A	Twin B
·Small PFO with left to right shunt.	·Cardiac anatomy not clearly defined.
·Two separate AV valves seen.	·A single AV valve with hypoplastic AV valve annulus.
·Adequately sized heart chambers.	·Small ventricle.
·No AV valve regurgitation.	·Large PDA (3mm diameter) with a left to right shunt.
·Small mid-muscular VSD (3 mm diameter) with a left to right shunt.	·Separate pulmonary artery and aorta.
·Adequately sized pulmonary artery and its branches.	·Most likely shared interventricular septum with Twin A.
·Small PDA (1mm diameter) with a left to right shunt	

#### 3.1.2 Abdominal ultrasound scan

This was done to get an overview of the abdominal organs; however, there were technical difficulties in obtaining diagnostic images. An initial USS showed separate livers and an unclear celiac trunk. CT angiography for elaboration of liver vasculature was therefore suggested.

#### 3.1.3 Whole body contrast enhanced CT scan

**Fig. 2:**
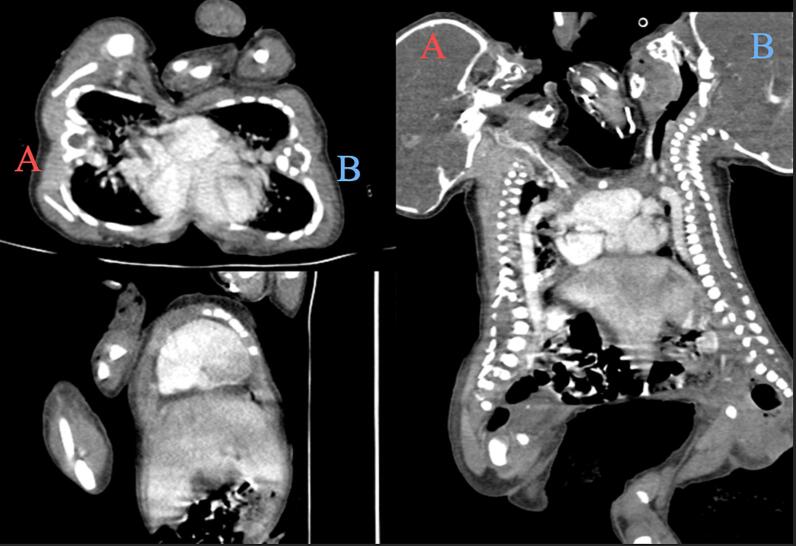
A contrasted CT scan in axial, coronal and sagittal views with twin A on the left and twin B on the right. It shows conjoined twins with fused chest and abdomen. The pericardium and heart are fused. There is enhancement of twin B’s heart and its vessels. The heart and liver are fused. Twin A has larger cardiac chambers than twin B.

**Fig. 3:**
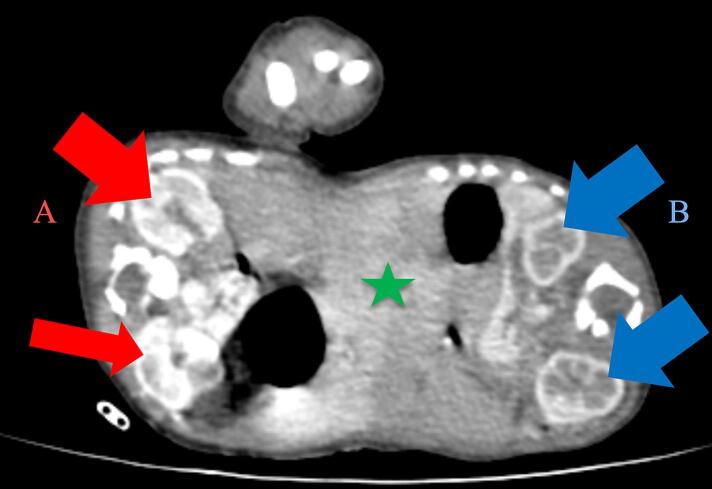
A contrasted CT scan in axial views with twin A on the left and twin B on the right. The liver (green star) is shared; however, the kidneys (red and blue arrows) are separate and normal.

**Fig. 4:**
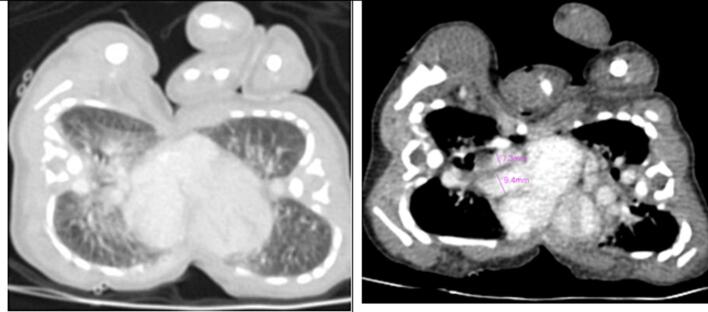
A contrasted CT scan in axial views with twin A on the left and twin B on the right. The lungs were separate; however, there is significant engorgement of twin A’s pulmonary vasculature in the lung window. The main pulmonary artery to aorta (MPA:A) ratio for twin A in the mediastinal window is 1.3, which falls in the normal range.

The initial contrast-enhanced CT scan provided intricate details, introducing contrast into Twin A only. Fusion from the upper chest to the umbilical area was observed, and the neonatal hearts exhibited an unclear cleavage between them, as illustrated in Fig. 2, Fig. 3, Fig. 4. Twin A's larger heart chambers were noted, with contrast crossing into Twin B. The pericardium separation was unclear, and the sternal components were not visualised. The shared liver, while evident, lacked clarity in the biliary system and vasculature. The distinctive left-sided thoracic aorta of twin A and the right-sided aorta of twin B led to the conclusive diagnosis of thoraco-omphalopagus conjoined twins with a shared heart, pericardium, and shared liver. This is illustrated in Fig. 2, Fig. 3, Fig. 4.

**Fig. 5:**
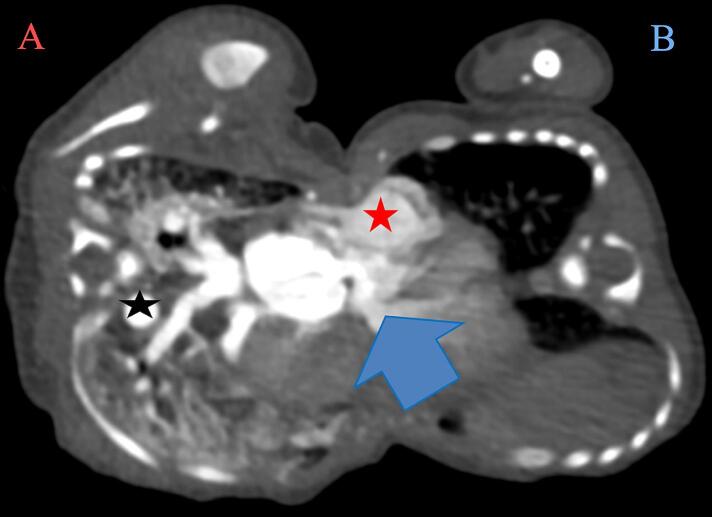
A contrasted CT scan in axial views with twin A on the left and twin B on the right. Twin A has a left-sided right ventricle (red star) with communication to the heart of twin B (blue arrow) and a right-sided descending aorta (black star). There is marked engorgement of the pulmonary vessels of twin A (increased lung parenchymal enhancement).

**Fig. 6:**
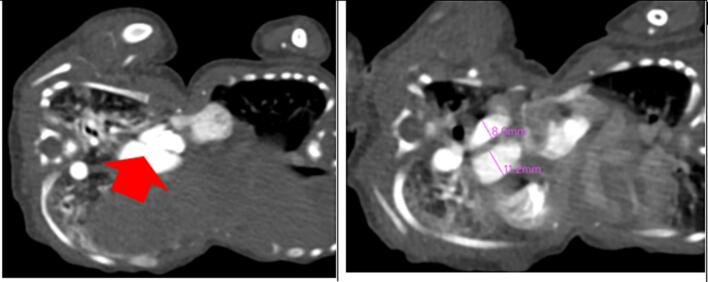
A contrasted CT scan in axial views with twin A on the left and twin B on the right. There is a primum atrial septal defect (red arrow) in the heart of twin A. The second image denotes an increased main pulmonary artery to aorta ratio (MPA:A) of 1.4 for twin A.

**Fig. 7:**
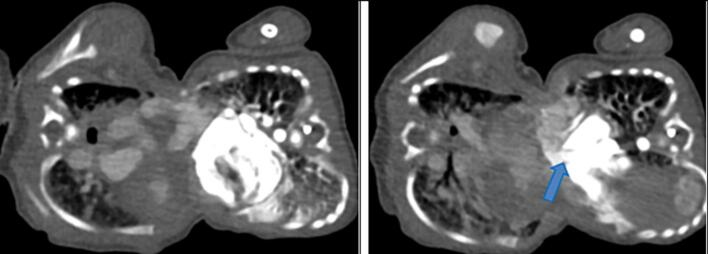
A contrasted CT scan in axial views with twin A on the left and twin B on the right. The heart chambers of twin B are malformed and small. A defect connects to twin A’s heart (blue arrow).

**Fig. 8:**
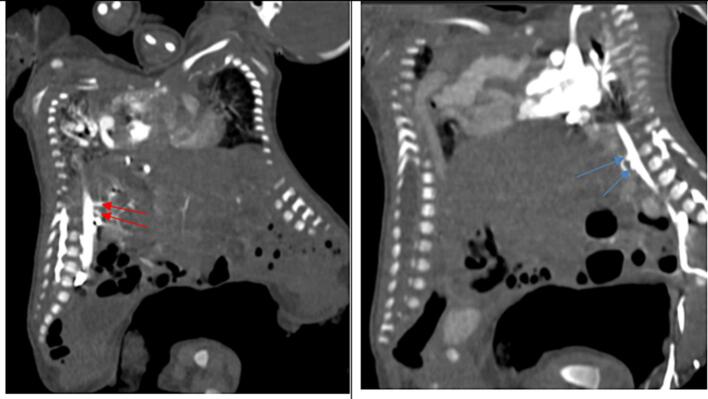
A contrasted CT scan in sagittal views with twin A on the left and twin B on the right. Twin A (red arrows) and twin B (blue arrows) have separate coeliac trunks and superior mesenteric arteries.

**Fig. 9:**
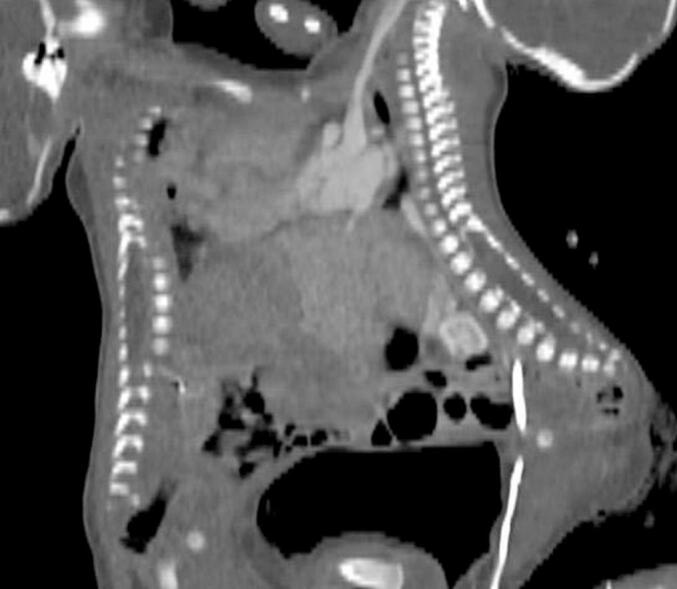
A contrasted CT scan in sagittal views with twin A on the left and twin B on the right in the delayed phase. It shows differential enhancement of the liver parenchyma of both twins.

A follow-up CT angiography (Fig. 5, Fig. 6, Fig. 7, Fig. 8, Fig. 9) performed two weeks later provided deeper insights. Separate contrast enhancement of Twin A revealed dextrocardia, a primum atrial septal defect, a right ventricle with a defect on the non-septal wall to Twin B with contrast crossing over, enlarged pulmonary arteries indicative of heart failure, and a right-sided descending aorta. Due to the right-sided aortic arch, dextrocardia, and left-sided large lobe of the liver, Twin A was suspected to have situs inversus. In contrast, Twin B exhibited a congenitally malformed heart, minimal contrast crossover, a left-sided descending aorta, and a separate liver seen in a delayed CT scan with a differential enhancement of the combined livers.

### 3.2 Follow up and monitoring

Advanced hemodynamic monitoring techniques such as functional echocardiography or blood flow measurements were not available in our setting. Neonatal assessment was primarily clinical, supported by basic monitoring tools, laboratory investigations and imaging.

During their stay on the ward, the babies were periodically reviewed by the pediatric surgery team, and their treatment was adjusted as needed. On the 5th day of life, a nutritionist assessed the twins, and they were started on expressed breast milk (EBM) at 25 mL for Twin A and 20 mL for Twin B. This was gradually increased over five days to 35 mL and 30 mL, respectively, by the 10th day.

On the 10th day of life, the twins developed fevers. Antibiotics were changed from intravenous (IV) cefotaxime to IV piperacillin-tazobactam. Complete blood count (CBC) and blood cultures were taken, with the CBC showing increasing trends in white blood cell (WBC) count.

On the 14th day of life, despite the fevers subsiding after the antibiotic switch, Twin A developed worsening respiratory distress. Further evaluation revealed basal crepitations in the lung bases. A cardiologist diagnosed heart failure and Twin A was started on IV furosemide and Digoxin.

By Day 18, their combined weight had dropped by 1 kg to 4.7 kg. A multidisciplinary team discussion on the 19th day of life was done to review the twins' status, including imaging findings, to plan further management.

### 3.3 Multidisciplinary team discussion

The multidisciplinary team included a social worker, nurses, pediatric surgeons, neonatologists, pharmacist, pediatric cardiac surgeons, radiologists, and anesthesiologists, who jointly participated in the case conferences and inpatient care planning.

During the discussion, the major contention revolved around the complex cardiac anatomy of the conjoined twins, with particular emphasis on the severe cardiac defects of Twin B. It was highlighted by pediatric cardiac surgeons that the intricate heart structure of Twin B posed a significant contraindication for surgical separation, and any attempt at separation should prioritize Twin A due to her more favorable cardiac anatomy, implying the potential need to “sacrifice” Twin B to save Twin A. This posed a complex ethical dilemma. Furthermore, the anesthesia team echoed the concern, stating that the abnormal cardiac function made survival post-separation highly improbable for both twins in the current setting. Additionally, the pediatric cardiologists suggested seeking technical support from overseas teams, given the lack of capacity within Uganda to manage such a complex case. The discussion also covered practical considerations, such as the use of CT scans over MRI for imaging and the challenges of adequately medicating both twins through a shared circulatory system.

The team concluded that the twins would not survive a separation procedure within the Ugandan setting, necessitating support from more equipped international teams. The cardiology team advised continuing with the heart failure medication for Twin A, who was exhibiting signs of congestive heart failure. Furthermore, the nutritionist was requested to review the feeding regimen of the twins to address the issue of weight loss. The team decided to explore options for external support by referring the twins to provide the best possible care for the twins, acknowledging the technical and logistical challenges they faced locally. The team also informed the family about the recommendations from this conference via a family conference.

### 3.4 Outcomes

Unfortunately, the twins passed away on Day 31, with heart failure suspected as the primary cause of death. Postmortem findings correlated with previous imaging, confirming a shared liver and complex cardiac anatomy. Examination revealed twins with a single umbilical cord, separate heads, necks, limbs, and gastrointestinal tracts, but a fused rib cage and diaphragms. They shared a pericardium and a complex congenital heart comprising three ventricles, four atria, and separate great vessels (two aortas and two pulmonary trunks). The liver was fused at the midline with two separate gallbladders, while other abdominal, genitourinary, and skeletal structures were separate and grossly normal. The cause of death was attributed to complications of the shared cardiac anatomy, with pulmonary congestion suggesting significant hemodynamic compromise.

## 4 Discussion

Conjoined twins, a rare phenomenon where genetically identical individuals are physically joined at birth, present complex medical challenges. Two primary theories explain their formation: Fission theory, suggesting incomplete embryonic division, and Fusion theory, which proposes that adjacent embryos fuse during development [17]. No consistent environmental or maternal risk factors have been confirmed, and reported associations (for example, an isolated hypothesis implicating griseofulvin) have not been reproducible in subsequent studies [18,19]. Likewise, recurrence in subsequent pregnancies is exceedingly rare and there is no clear evidence to recommend routine genetic counseling for most families beyond standard antenatal care [20].

Conjoined twins are categorized into three major groups based on their union: ventral, dorsal, and lateral unions [11,21]. These groups are further classified by the most prominent point of conjunction: skull (cephalopagus), thorax (thoracopagus), abdomen (omphalopagus), pelvis (ischiopagus), sacrum (pygopagus), and back (rachipagus) [14,22]. Ventral fusion is the most common type, with the thorax being the predominant fusion site [21]. This typically results in thoracopagus or thoraco-omphalopagus, with the fusion of thoracic and abdominal cavities, as seen in this case.

Most cases of conjoined twins are diagnosed prenatally, often through the use of low-cost imaging modalities such as ultrasound scans [23]. Early diagnosis provides an opportunity for prenatal counseling, delivery planning, and postnatal care [14,22]. However, in our case, antenatal diagnosis was prevented due to power shortages and technical issues with the ultrasound machine at the peripheral unit that referred the twins. This scenario is not uncommon in low-resource settings [24]. In the absence of antenatal diagnosis, the medical team is often faced with an unexpected situation at birth.

This case underscores the importance of routine antenatal care and timely obstetric ultrasound for the early detection of major fetal anomalies [25]. In settings such as refugee settlements where antenatal attendance and imaging access are limited, targeted strategies are needed, including training and sensitization of midwives, obstetricians, and radiographers to recognise and refer suspected high-risk pregnancies, strengthening referral pathways to maternal-fetal or high-risk obstetric units, and partnering with humanitarian agencies to expand outreach and portable ultrasound services [26]. Establishing clear referral algorithms and teleconsultation links with tertiary centres can facilitate earlier multidisciplinary planning, informed counseling, and timely transfer when necessary [27].

Current recommendations suggest that viable twins at term should be delivered by cesarean section at 36 to 38 weeks (C-section) to minimize risks associated with vaginal delivery, such as avulsion injuries, internal hemorrhage, intrapartum demise, shoulder dystocia, and uterine rupture [8,9]. For preterm conjoined twins with demise and maceration, spontaneous vaginal delivery (SVD) is preferred if possible [12,28]. In our case, the twins were born alive via an emergency C-section due to a footling presentation.

Our case reflects the common occurrence of cardiac fusion in thoraco-omphalopagus twins, with an unclear cleavage between neonatal hearts and varying heart chamber sizes [29,30]. There was a non-septal wall defect of the ventricular wall of twin A with crossover of contrast through the defect into the heart of twin B. Coupled with enlargement of the pulmonary vessels of twin A and the malformed heart of twin B, this indicates that twin A was meeting the cardiovascular demands of both twins. This also explains why twin A was going into heart failure. This unique feature of this case warranted a cardiologist's review of the twins.

The dextrocardia and left-sided thoracic aorta in twin A and the right-sided thoracic aorta in twin B highlight the critical role of angiography to delineate the vascular anatomy in surgical planning. [31,32] Twin A was suspected of having situs inversus with dextrocardia, a right-sided aortic arch, and a larger lobe of its liver to the left. Our case mirrors the commonality of the shared liver in thoraco-omphalopagus twins, with a poorly defined biliary system impacting postnatal management. This aligns with existing literature recommendations, emphasising the need for a detailed evaluation of the liver [6,11].

In our case, the use of a delayed CT scan, revealing separate liver enhancement and differential enhancement of combined livers, aligns with literature suggestions for obtaining dynamic information. Sequential imaging, as exemplified in our case, is acknowledged in the literature for guiding surgical decisions through a comprehensive understanding of evolving anatomical features. The complex cardiac fusion, in our case, necessitated multidisciplinary expertise in surgical planning. Detailed vascular mapping, as emphasized in our case and literature, becomes paramount for determining the optimal surgical approach [14,32].

Survival rates in thoraco-omphalopagus conjoined twins, as seen in our case, are intricately linked to organ sharing and overall health. Successful separation and meticulous postoperative care significantly impact long-term outcomes. Our case in a refugee setting highlights challenges in access to prenatal care and advanced imaging. Multidisciplinary teams must adapt to resource constraints, emphasising the importance of creative solutions in these challenging environments.

Parents face difficult decisions regarding the surgical intervention. Ethical discussions involving quality of life, survival, and individual autonomy become pivotal, emphasising the need for open and compassionate communication within the multidisciplinary team and with the parents.

Management of conjoined twins requires a MDT approach involving various hospital teams, including pediatric surgeons, nurses, radiologists, anesthesiologists, and pharmacists [10,15]. The comprehensive imaging protocols and MDT discussions act as a foundational framework for the preoperative planning and successful surgical separation detailed in this case report of conjoined twins [13]. During these discussions, medical issues, challenges, and the aberrant physiology of cross-circulation necessitated adjustments in dosing. Key themes include planning for delivery, surgical planning, postoperative management, and resuscitation. Simulation drills for resuscitation, delivery, oxygen delivery, and surgical techniques using 3D models and dummies can also be beneficial [15].

In our case, the MDT discussion highlighted the complexity of the conjoined twins, especially the challenging cardiac structure of Twin B which created an ethical dilemma of prioritizing one twin over the other was considered. In this case antenatal diagnosis would have allowed earlier discussion of the risks and potential options, including whether a planned strategy to favour the twin with more favorable anatomy could have been ethically and clinically justified. However, at the time of postnatal review Twin A was already exhibiting clinical and radiologic features of cardiac failure, which limited the viability of any operative strategy that might have required sacrificing Twin B to attempt to save Twin A. We therefore emphasize that timing of diagnosis is critical, and that ethical deliberations in such cases must balance prognosis, parental values, and the resource constraints of the local setting. Institutional ethics consultation, clear documentation of family discussions, and culturally sensitive counseling are recommended components of care when these dilemmas arise about the possibility of sacrificing one twin to save the other if separation was attempted [33,34].

## 5 Conclusion

This case of thoraco-omphalopagus conjoined twins in a 27-year-old refugee underscores the complexity and uniqueness of this rare medical phenomenon. Compared with existing literature, the detailed imaging findings reveal nuances in cardiac fusion, vascular anatomy, and liver sharing that are crucial for surgical planning and prognostic considerations. The clinical implications highlight the multidisciplinary challenges associated with these cases, necessitating expertise in detailed vascular mapping and careful postoperative care to influence outcomes. In resource-limited settings, such cases also bring forward profound ethical considerations in separation, particularly when antenatal diagnosis is missed and treatment options become limited. Strengthening access to regular antenatal care, timely obstetric ultrasound screening, and referral to high-risk maternal–fetal medicine units is essential for early decision-making and counseling. Targeted community sensitization and outreach strategies, especially in refugee and underserved populations, are critical to ensuring that high-risk pregnancies are identified and managed in specialist centers.

## Consent for publication

Written informed consent was obtained from the patient for publication of this case report and any accompanying images.

## Ethical approval

Ethical approval is not required for case reports at our local hospital IRB.

## Funding

There are no funding sources for this case report.

## Author contribution

Conceptualization: Brian Kasagga, Roy Clark Basiimwa.

Methodology: Brian Kasagga, Yasin Ssewanyana, Rita Nassanga.

Investigation: Stella Nimanya, Yasin Ssewanyana, Brian Kasagga.

Writing – Original Draft: Brian Kasagga, Roy Clark Basiimwa.

Writing – Review & Editing: John Sekabira, Roy Clark Basiimwa, Brian Kasagga.

Supervision: John Sekabira.

## Guarantor

Brian Kasagga.

## Research registration number

NA.

## Conflict of interest statement

The authors declare that they have no competing interests.

## Data Availability

For this case report, clinical information was obtained from patient files. On request, more details are available, but only in compliance with the hospitals' privacy rules.
